# Outcomes of patients with continuous flow left ventricular assist device undergoing emergency endovascular treatment for atraumatic bleeding

**DOI:** 10.1186/s42155-019-0085-x

**Published:** 2019-12-09

**Authors:** Valentina Bernardinello, Giulio Barbiero, Michele Battistel, Caterina Dengo, Roberto Stramare, Giulio Folino, Jonida Bejko, Massimiliano Carrozzini, Vincenzo Tarzia, Gino Gerosa, Tomaso Bottio

**Affiliations:** 1 Department of Medicine DIMED, Institute of Radiology, Via Giustiniani 2, 35128 Padova, Italy; 2 Radiology, M. Bufalini Hospital, Viale Ghirotti 286, 47521 Cesena, Italy; 3 Department of Cardio-Thoracic Vascular Sciences and Public Health, Cardiac Surgery, Via Giustiniani 2, 35128 Padova, Italy

## Abstract

**Introduction:**

Severe spontaneous bleeding is a significant complication in patients with continuous flow left ventricular assist devices; there is little evidence on endovascular treatment to support its use.

**Materials and methods:**

We observed seven patients (five men, two women, age 43–67 years) with continuous flow left ventricular assist devices on antiaggregant/coagulant therapy, admitted to our hospital for uncorrectable symptomatic anemia; CT-angiography and diagnostic angiography confirmed the presence of atraumatic arterious bleeding from the gastrointestinal tract (six patients), from the intercostal artery and from the bronchial tree (one patient).

**Results:**

All patients where successfully treated via an endovascular approach with superselective embolization of the involved arterial branches with coils and particles.

**Conclusion:**

Spontaneous atraumatic bleeding is a frequent complication in patients with continuous flow left ventricular assist devices; endovascular treatment represents a promising alternative to the surgical approach as it is less invasive, easily repeatable and associated to a reduced procedural risk.

## Introduction

In recent years, the growing number of patients with advanced heart failure, the limited availability of donor organs and the continuous improvements of the devices have led to a marked acceleration in the use of left ventricular assist devices (LVADs) as bridge to transplant, for destination therapy (ie, permanent support in patients who are not candidates for transplant), but also as a bridge to myocardial recovery (ie, temporary support in patients in whom the heart is expected to recover after an acute, reversible cardiac injury) (Kirklin et al. 2017; Carrozzini et al. 2019a, 2019c; Carrozzini et al. 2018; Todisco et al. 2018).

Last generation of continuous-flow left ventricular assist devices (CF-LVADs) has demonstrated enhanced reliability and durability, leading to satisfactory long-term survival and improved quality of life of patients for extended periods of support. Yet, their use is associated to a risk of complications, including infection, device malfunction, arrhythmias, right ventricular failure, thromboembolic disease, and post-operative nonsurgical bleeding (Gurvits et al. 2017).

Spontaneous atraumatic bleeding, which in most patients originates from the gastrointestinal tract, is a significant complication in patients carrying CF-LVADs and is the most common reason for readmission; it is estimated that this complication involves about half of patients within 1 year of CF-LVAD placement (Mehta et al. 2015). That is due to the combination of antiplatelet and vitamin K antagonist therapy, activation of fibrinolytic pathway, acquired von Willebrand factor deficiency, and tendency to develop angiodysplasias and gastrointestinal arteriovenous malformations due to increased rotary speed of the pump (Gurvits et al. 2017; Sieg et al. 2017; Kang et al. 2017).

In order to generate evidence to support endovascular treatment of this patient cohort, we show a case series of our experience in treatment of this condition via an endovascular approach.

## Materials and methods

We reviewed all consecutive patients carrying CF-LVAD who were hospitalized in our centre for severe anaemia between April 2014 and February 2019. The indication for the implantation of CF-LVAD was detailed.

All patients at the time of the bleeding were on antiplatelet and/or anticoagulant therapy (vitamin K antagonist or new oral anticoagulant NOAC).

Arterial access was conducted by ultrasound guidance, after identification of the common femoral artery: in these patients it was obviously not possible to find the arterial pulse due to the presence of continuous flow stimulated by the device.

In all patients, after diagnostic angiography, superselective catheterization of the involved arterial branches was performed by the use of micro-catheters (Progreat, Terumo, Tokyo, Japan), with subsequent embolization with permanent particulate embolization material (Contour emboli, Boston Scientific, Natick, MA, USA), between 150 μm and 710 μm) and microcoils (Tornado, Cook, Bloomington, IN, USA and Concerto, ev3, Covidien, Playmouth, MA, USA), or with resorbable particulate embolizing material (Spongostan, Johnson & Johnson Ethicon Inc., New Brunswick, NJ, USA).

In all cases we have chosen to leave the arterial introducer in place at the end of the procedure to allow more rapid intervention in the event that re-treatment was necessary; after 24–72 h from the procedure we removed the arterial introducer by performing manual compression on the access site.

All procedures were conducted under local anesthesia (lidocaine 2%), with non-invasive monitoring of the vital signs.

## Results

We collected 7 consecutive patients (5 men and 2 women; median age, 57 years; age range, 43–67 years) with CF-LVAD which were referred to our centre for embolization of atraumatic active arterial bleeding from April 2014 to February 2019. Mean time from surgery was 38 days (range, 8–113 days).

Indication for implantation of CF-LVAD was severe ischemic heart failure (6 cases) and primary dilated cardiomyopathy (1 case).

Antiplatelet and/or anticoagulant therapy type and dose, and coagulation parameters were specified in Table 1.

**Table 1:** Patients cohort with antithrombotic therapy and coagulative profile at the time of bleeding (* second bleeding)

PATIENT N°	AGE (years)	SEX (M/F)	THERAPY (drug (dose))	International Normalized Ratio (INR)	Prothrombin Time (PT) (%)	Activated Partial Thromboplastin Time (APTT) (s)
1	67	M	Warfarin	1,13	64	43
2	43	M	Fondaparinux (5 mg/day)	1,56	38	40
3	49	M	Aspirin (160 mg/day) and Fondaparinux (5 mg/day)	1,28	52	24
4	59	F	Aspirin (100 mg/day) and Fondaparinux (2,5 mg/day)	1,10	67	26
5	63	M	Aspirin (100 mg/day) and Fondaparinux (2,5 mg/day)	1,16	79	28
6	65	M	Fondaparinux (2,5 mg/day)	1,04	94	38
7	53	F	Fondaparinux (2,5 mg/day)	1,09	84	32
				1,05*	92*	39*

* indicates the coagulation values of patient 7 during the second bleeding episode

Six patients presented spontaneous bleeding originating from the gastrointestinal tract, while one patient had intercostal and bronchial bleeding.

**Fig. 1:**
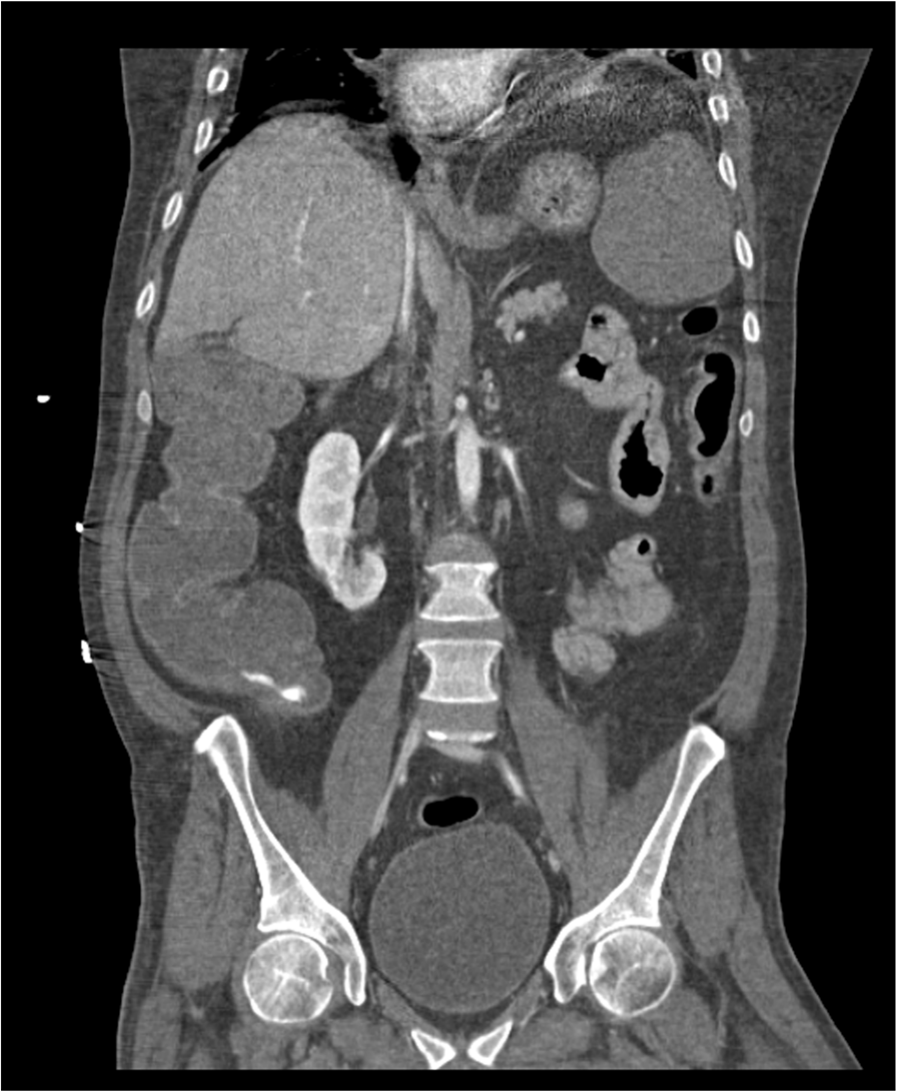
Coronal CT-angiography image shows active arterial bleeding in the cecum

**Fig. 2:**
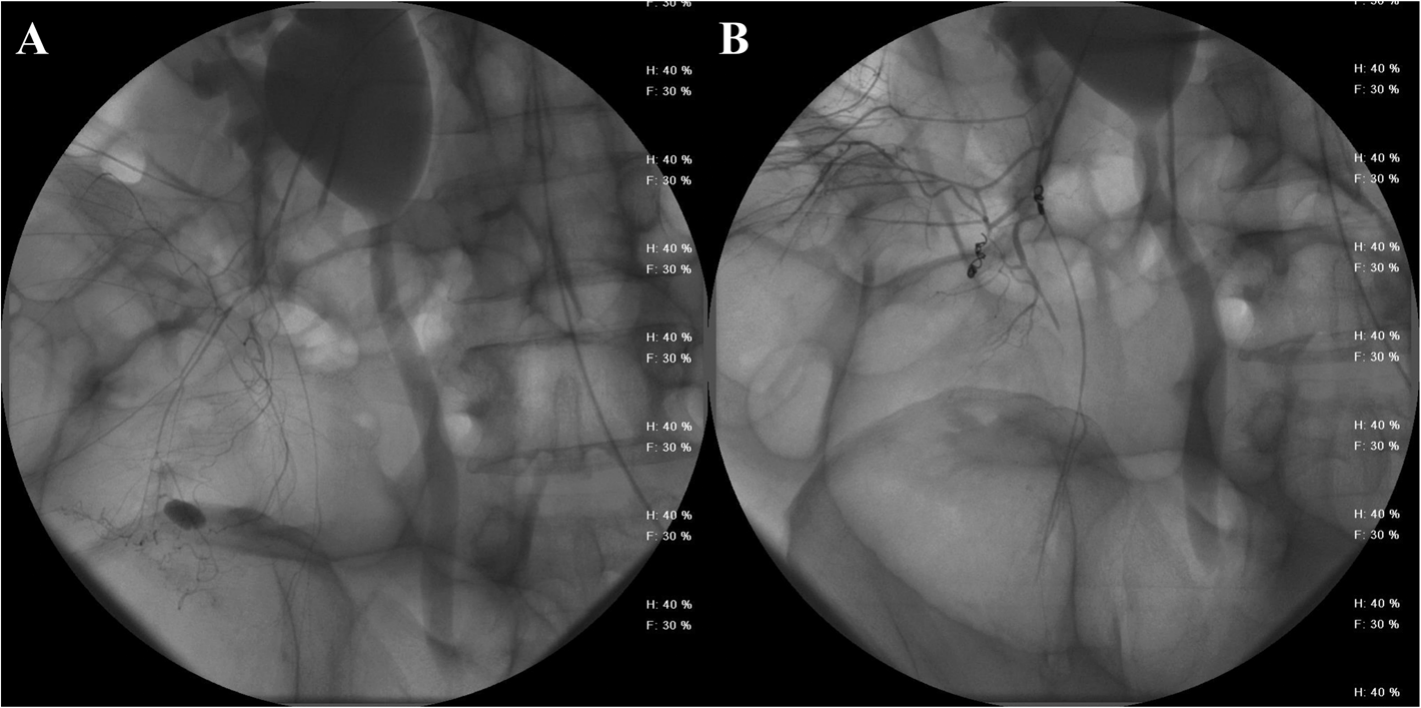
(**a**) Diagnostic angiography confirmed the presence of bleeding from branches of the superior mesenteric artery directed to the cecum. (**b**) Angiography image shows the absence of active bleeding at the end of the procedure

**Fig. 3:**
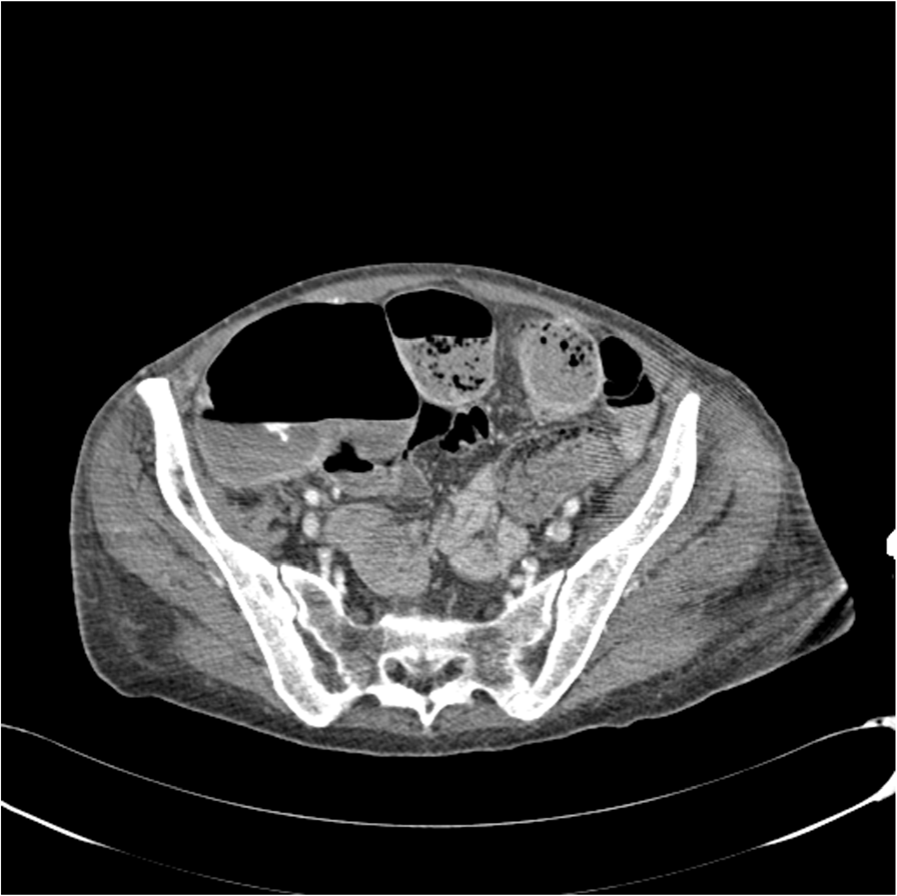
Axial CT-angiography image shows active arterial bleeding in the ascending colon

**Fig. 4:**
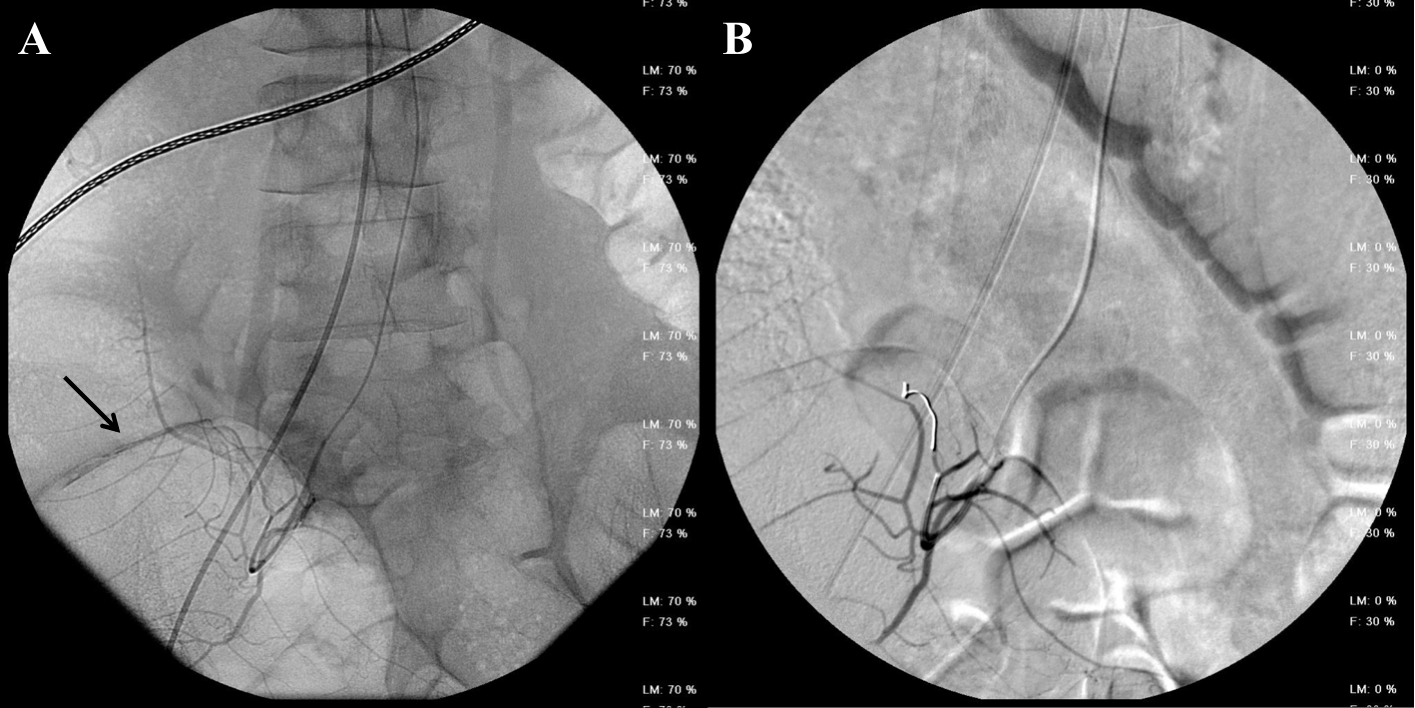
(**a**) Diagnostic angiography confirmed the presence of bleeding from branches of the superior mesenteric artery directed to the ascending colon (black arrow). (**b**) Angiography image shows the absence of active bleeding at the end of the procedure

**Fig. 5:**
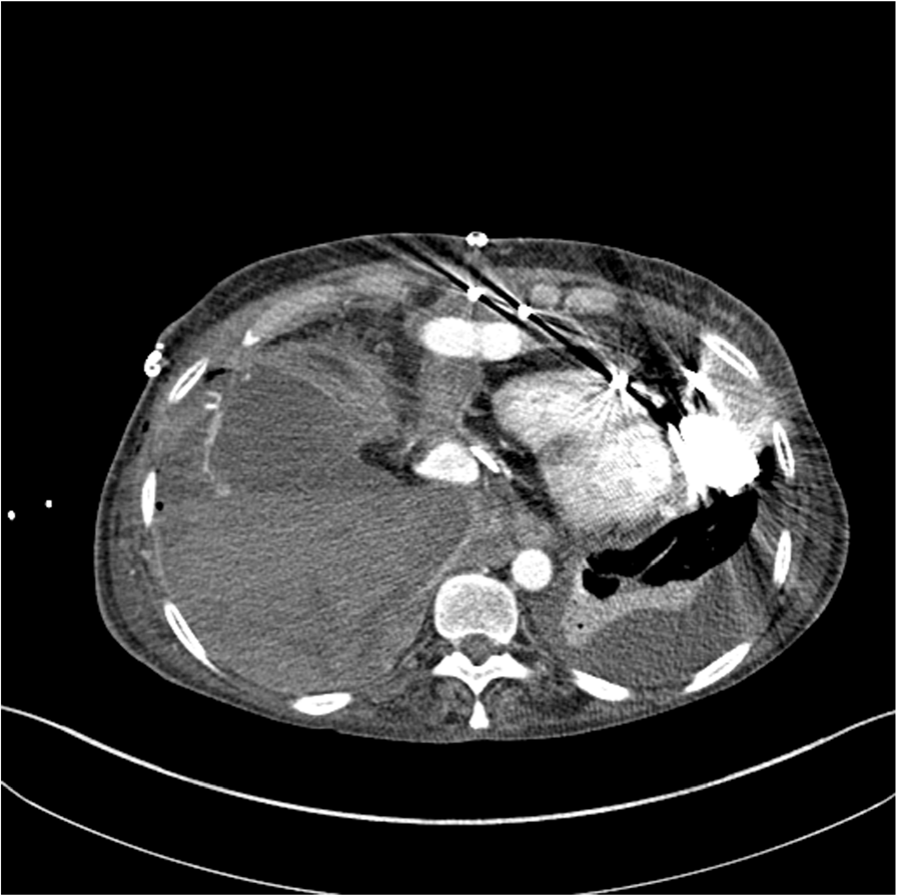
Axial CT-angiography image shows active arterial bleeding in the thorax from the IX intercostal right artery

**Fig. 6:**
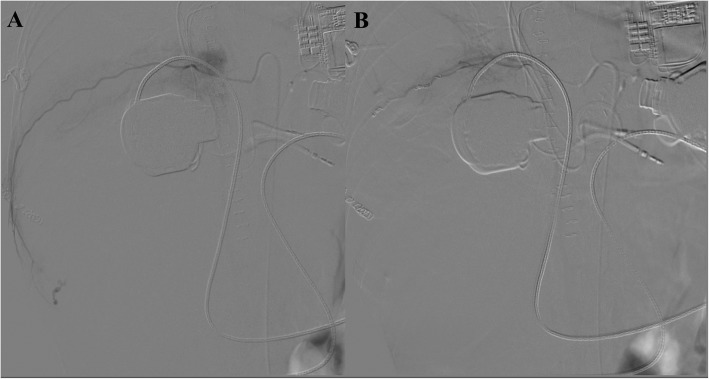
(**a**) Diagnostic angiography confirmed the presence of bleeding from the IX intercostal right artery. (**b**) Angiography image shows the absence of active bleeding at the end of the procedure

All patients presented acute anaemia and underwent CT-angiography with the diagnosis of atraumatic active arterial bleeding. Diagnostic angiography, performed after CT, confirmed the presence of bleeding from branches of the superior mesenteric artery directed to the ascending colon-cecum (3 cases) (Figs. 1, 2, 3, 4), to the last ileal loop (1 case) and to the descending colon (1 case), from branches of the pancreatic-duodenal artery (1 case) and from the IX intercostal right artery (1 case) (Figs. 5, 6).

Different embolization matherials were used (particulate and microcoils in 6 cases; resorbable particulate in 1 case).

After the procedure only one patient needed a re-treatment after three days, with prompt resolution of the bleeding, while the others remained hemodynamically stable.

One patient had a new episode of arterial bleeding in another location (bronchial artery) twenty-one days after the first embolization, which was also successfully treated by superselective embolization.

None of the examined patients presented post-procedural complications related to the embolization procedure.

Currently, of these seven patients, two were transplanted, four were in stable condition waiting for transplant, and one died due to causes unrelated to the interventional procedure.

## Discussion

Severe atraumatic bleeding, especially of gastrointestinal origin, is an important and frequent complication of CF-LVAD therapy; the management requires a multidisciplinary approach. The history and physical exam should suggest the initial site of bleeding: patients with hematemesis or melena should be considered to have upper gastrointestinal bleeding and should undergo upper endoscopy (Gurvits et al. 2017), while patients with hematochezia can be investigated for suspected lower gastrointestinal bleeding, with the initial examination being colonoscopy (Carrozzini et al. 2019b). When endoscopy is inconclusive, other exams are necessary. CT can be used to identify LVAD-related bleeding complications and it is often diagnostic also for non-gastrointestinal bleeding, such as pericardial, pleural, abdominal wall, retroperitoneal and intracranial hemorrhage (Carrozzini et al. 2019a).

The management of gastrointestinal bleeding in patients with CF-LVAD is predominantly medical, with reduction of anticoagulation, and administration of concentrated red blood cells, octreotide and proton-pump inhibitors, although the data regarding their overall efficacy remain sparse (Sieg et al. 2017). If these treatments are not effective, surgery is considered, even if the surgical and anesthetic risk of these patients is considerably increased by their coagulation defects and by the presence of important comorbidities (Guha et al. 2015). Endovascular therapy is obviously less invasive with less anesthesia risk, and can be performed on patients unfit for surgery, as is often the case in those with cardiac disorders; moreover it is a repeatable and poorly invasive procedure.

As previously mentioned, these patients have a high risk of bleeding due to antithrombotic therapy and other pathophysiological mechanisms due to the continuous flow of the cardiac device; antithrombotic therapy can be corrected if overdosed, but usually not interrupted. This obviously makes the endovascular approach advantageous compared to the surgical one, because it requires just a small arteriotomy (4 or 5 French in our experience) without increasing the risk of further anaemia via access site wound.

The inability to suspend anticoagulant therapy affects the choice of embolics agents as some (eg coils) may not be as effective as others (eg gelfoam, NBCA); in most cases, in fact, we have chosen to combine the injection of permanent particulate embolization material with microcoils.

Few studies are available about the effectiveness of interventional radiology procedures in CF-LVAD patients: a case of Morito and others (Morito et al. 2014) reports the successful endovascular treatment of a patient with a cerebral hemorrhage caused by ruptured cerebral aneurysms, detected by angiography; the clinical study by Metha et al. (Mehta et al. 2015) reports that patients with LVADs presenting with gastrointestinal bleeding have fewer successful embolizations and a higher rate of clinical failure than the control group of patients.

In our experience the diagnostic angiography, performed after a positive contrast enhancement-CT, identified the bleeding site in 100% of cases. In 6/7 (85,7%) patients the embolization procedure performed immediately after the diagnostic angiography was therapeutic, with normalization of hemoglobin values and rapid recovery; only 1/7 (14,3%) patient was again subjected to embolization 21 days later, with a successful procedure. No patient presented complications.

Of course there are potential problems with endovascular approach. At the end of the procedure it is possible that, due to the anticoagulation, difficulties occur in the closure of the arteriotomy with the consequent need for closing devices: however in our experience the small caliper of the arteriothomy, a prolonged manual compression on the access site and the immobilization of the limb for the next 24 h were sufficient for the closure of the arteriotomy. In the case of intermittent bleeding it can happen that during the arteriography the spread of contrast medium is not evident: in our series the superselective arteriography of the branches previously identified at the CT-angiography always identified the sites of the bleeding, but in the case where this eventuality occurs we believe that a multidisciplinary approach with clinicians and surgeons is needed before proceeding with prophylactic embolization. Finally, as in all cases of gastrointestinal embolization, one of the most feared complications is the bowel infarction with subsequent need for surgical resection, further complicated in these cases by the high risk of bleeding and anesthesiology: fortunately this did not occur in our patients; however, we believe that the superselective embolization of the only branches responsible for bleeding reduces this risk.

Certainly not all sources of bleeding in CF-LVAD patients are susceptible to endovascular treatment. Mediastinal bleeding often occurs in the first week after implantation of the device, in most cases due to surgical complications (Nascimbene et al. 2016) and for this reason they are better controlled with the surgical approach; non-surgical bleeding, instead, usually arises later, in distant site bleeds and at the site of arteriovenous malformations: these are the cases in which the endovascular approach should be considered as the first line treatment.

## Conclusion

In conclusion, our opinion is that the urgent endovascular treatment of spontaneous bleeding in patients with continuous-flow left ventricular assist devices is an effective life-saving procedure and a valid alternative to more invasive and risky procedures, especially if carried out by expert interventional radiologists and after adequate selection of patients through preliminary CT study. More studies with a greater number of patients are needed to validate the technique.

## Data Availability

The datasets used and/or analysed during the current study are available from the corresponding author on reasonable request.

## Abbreviations

CF-LVAD: continuous-flow left ventricular assist devices
LVAD: left ventricular assist devices
NOAC: new oral anticoagulant
